# Sexually dimorphic transcriptional programs of early-phase response in regenerating peripheral nerves

**DOI:** 10.3389/fnmol.2022.958568

**Published:** 2022-08-02

**Authors:** Andrei V. Chernov, Veronica I. Shubayev

**Affiliations:** ^1^Department of Anesthesiology, University of California, San Diego, San Diego, CA, United States; ^2^VA San Diego Healthcare System, San Diego, CA, United States

**Keywords:** sexual dimorphism, peripheral nerve, axotomy, nerve regeneration, RNA-seq

## Abstract

The convergence of transcriptional and epigenetic changes in the peripheral nervous system (PNS) reshapes the spatiotemporal gene expression landscape in response to nerve transection. The control of these molecular programs exhibits sexually dimorphic characteristics that remain not sufficiently characterized. In the present study, we recorded genome-wide and sex-dependent early-phase transcriptional changes in regenerating (proximal) sciatic nerve 24 h after axotomy. Male nerves exhibited more extensive transcriptional changes with male-dominant upregulation of cytoskeletal binding and structural protein genes. Regulation of mRNAs encoding ion and ionotropic neurotransmitter channels displayed prominent sexual dimorphism consistent with sex-specific mRNA axonal transport in an early-phase regenerative response. Protein kinases and axonal transport genes showed sexually dimorphic regulation. Genes encoding components of synaptic vesicles were at high baseline expression in females and showed post-injury induction selectively in males. Predictive bioinformatic analyses established patterns of sexually dimorphic regulation of neurotrophic and immune genes, including activation of glial cell line-derived neurotrophic factor Gfra1 receptor and immune checkpoint cyclin D1 (Ccnd1) potentially linked to X-chromosome encoded tissue inhibitor of matrix metallo proteinases 1 (Timp1). Regulatory networks involving Olig1, Pou3f3/Oct6, Myrf, and Myt1l transcription factors were linked to sex-dependent reprogramming in regenerating nerves. Differential expression patterns of non-coding RNAs motivate a model of sexually dimorphic nerve regenerative responses to injury determined by epigenetic factors. Combined with our findings in the corresponding dorsal root ganglia (DRG), unique early-phase sex-specific molecular triggers could enrich the mechanistic understanding of peripheral neuropathies.

## Introduction

The peripheral nervous system (PNS) displays strong regenerative potential compared to the brain and spinal cord. Substantial knowledge of mechanisms involved in PNS injury has been acquired using rodent models of sciatic nerve axotomy originally described by Augustus Waller in 1850 (Fawcett and Keynes, 1990; Stoll et al., 2002). The success of sensory recovery after PNS injury depends on molecular remodeling ensuing within 24 h post-axotomy in the proximal nerve segment (Gordon et al., 2003; McDonald et al., 2006; Zochodne, 2012) coordinated with an extensive transcriptional response within neuronal somas in the dorsal root ganglia (DRG) (McDonald et al., 2006; Chernov and Shubayev, 2021). Upon an initial die-back toward DRG, the proximal axotomized axons form axonal sprouts that mature into growth cones enriched in the cytoskeletal framework that drives axons to reestablish functional connections with the end organ (Lundborg, 1987; Burnett and Zager, 2004; Radtke and Vogt, 2009). The transcriptional landscape of the damaged PNS is enriched by axonally trafficked coding and non-coding (nc) RNAs (Cavalli et al., 2005; Avraham et al., 2021).

Axonal regrowth in the damaged PNS depends on trophic, immune, metabolic, and structural support of Schwann cells (Jessen and Mirsky, 2016, 2021; Merrell and Stanger, 2016; Milichko and Dyachuk, 2020), forming immediate partnerships with axons (McDonald et al., 2006). Schwann cells undergo extensive phenotypic transformation within 24 h post-injury in preparation for mitosis and alignment into bands of Büngner (Jessen and Mirsky, 2016, 2021; Merrell and Stanger, 2016; Milichko and Dyachuk, 2020). Schwann cells produce and deposit into basal lamina a plethora of growth-permissive and -inhibitory extracellular matrix (ECM) proteins and proteoglycans to guide axonal growth, such as laminin and chondroitin sulfate proteoglycans (McDonald et al., 2006). These activities are partly controlled by ECM-degrading matrix metalloproteinase (MMP)/ADAM families that reciprocally coregulate the ECM network with cytokines, chemokines, trophic factors, and adhesion molecules (Chattopadhyay and Shubayev, 2009; Liu et al., 2010, 2015; Kim et al., 2012; Chernov and Shubayev, 2021).

Sex is emerging as a key biological variable in models of PNS injury, as certain neuropathic states exhibit sex-specific prevalence, incidence, mechanisms, and clinical presentation (Unruh, 1996; Greenspan et al., 2007; Fillingim et al., 2009; Mogil, 2012; Sorge and Totsch, 2017; Boerner et al., 2018). Sexual dimorphism in the transcriptional landscape of adult PNS and its response to injury has been shown by RNA-sequencing (RNA-seq) analyses (North et al., 2019; Ray et al., 2019; Stephens et al., 2019; Chernov et al., 2020; Paige et al., 2020; Ahlström et al., 2021). Sex-related differences in the axon elongation (Kovacic et al., 2004) could potentially be attributed to metabolic, immune, neuroendocrine, and sex chromosome-related genetic programs that have shown sex-specific regulation in the damaged PNS (North et al., 2019; Ray et al., 2019; Stephens et al., 2019; Chernov et al., 2020; Mecklenburg et al., 2020; Paige et al., 2020; Tavares-Ferreira et al., 2020; Ahlström et al., 2021). Unique to females, the chromosome-wide epigenetic inactivation of one of the two X chromosomes (Xi) regulates the normal and aberrant activity of X-linked immunity-related genes (Bianchi et al., 2012), including that in DRG at 24 h after sciatic nerve axotomy (Chernov and Shubayev, 2021). Whether the corresponding regenerating nerve segments exhibit sexual dimorphism in response to PNS axotomy remains unknown.

Using high-depth RNA-seq, the present study identified sex-specific early-phase transcriptional changes in protein-coding and ncRNAs in regenerating (proximal) nerves at 24 h after sciatic nerve axotomy in male and female mice. Applying predictive bioinformatics, we determined unique signaling events related to regenerative, trophic, metabolic, and sex chromosome-linked systems.

## Results

**FIGURE 1:**
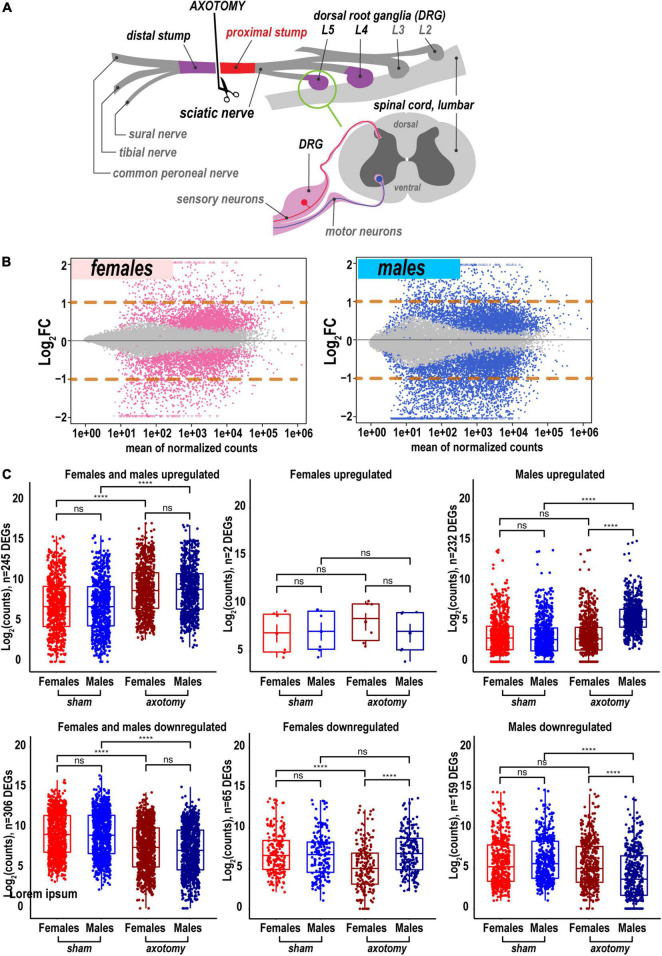
Global transcriptional changes in proximal nerve 24 h post-axotomy lead to a larger number of increased transcripts in males. **(A)** A schematic of the sciatic nerve axotomy followed by ipsilateral proximal stump tissue analysis. **(B)** MA plots display log_2_FC and mean counts normalized using *DESeq2* and adaptive t-prior *apeglm* method. The left and right panels show up- and downregulated genes in females (red dots) and males (blue dots), respectively. Dots indicate differentially expressed genes (DEGs) with P_adj_ < 0.1. Orange dashed lines show log_2_FC thresholds above 1 or below –1. **(C)** Scatter plots of significant up- and downregulated DEGs in females (light and dark red dots) and males (light and dark blue dots). Light and dark color intensity indicate DEGs in sham or axotomy samples (*n* = 6 mice/group, 2 mice/sample (pooled), 3 sample/group), respectively. DEGs (P_adj_ < 0.1) were filtered by log_2_FC either above 1 or below –1 (two-fold change or larger). Dots on the Y-axis correspond to the absolute gene expression measured as normalized mean counts following logarithm transformation of gene counts [log_2_(counts)] by the *normTransform* method in *DESeq2*. Specific expression data for DEGs is available in Supplementary Table 1 and the GEO repository (accession numbers GSE182713 and GSE182709). Pairwise comparisons were made using the analysis of variance (ANOVA) with the Tukey *post hoc* test: ****, *p* ≤ 0.00005; ns, not significant.

To determine the sex specificity of an early-phase PNS regenerative transcriptional program, sciatic nerve axotomy or sham operation was conducted in female and male mice. At 24 h post-axotomy, regenerating (proximal segment) tissues (Figure 1A) were subjected to whole-genome transcriptomics analysis by high-depth RNA-seq (Supplementary Figure 1, n = 6 mice/group, 2 mice/sample (pooled), 3 sample/group). At 24 h after the sham operation, nerve tissues were assessed for transcriptomic signatures without injury. Transcriptomics of the respective DRG tissues in the same animal cohorts was reported earlier (Chernov and Shubayev, 2021).

### Males exhibited more extensive transcriptional changes in regenerating sciatic nerve

A total of 25,788 genes were detected by RNA-seq in the proximal nerve stumps, including 2,553 differentially expressed genes (DEGs, Supplementary Table 1) that conformed to the significance criteria (adjusted p-values (P_adj_) < 0.1) and the expression fold change (FC) filter (log_2_FC > 1 or log_2_FC < –1, Figure 1A). In summary, more unique, highly expressed up-regulated DEGs (*n* = 232) were identified in males relative to females as presented on MA and scatter plots (Figures 1B,C). In addition, 65 and 159 DEGs were downregulated in female and male nerves, respectively.

### Sexually dimorphic gene regulation in injured nerves

**FIGURE 2:**
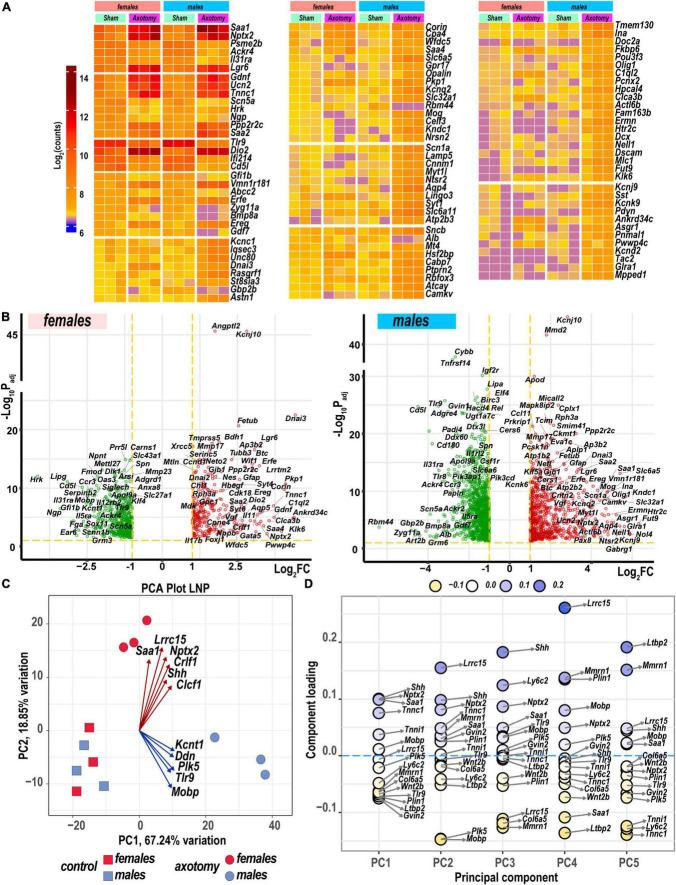
Nerve axotomy induced distinct sexually dimorphic regulation of differentially expressed genes (DEGs) in female and male mice. **(A)** Hierarchical clustering plot of 300 most significant upregulated DEGs (log_2_FC > 1, P_adj_ < 0.1, *n* = 6 mice/group, 2 mice/sample (pooled), 3 sample/group). Heatmap colors correspond to mean log_2_(counts)—Blue, yellow, and red – low, medium, and high expression, respectively. DEGs were sorted by log_2_FC. **(B)** Significant DEGs in female (left panel) and male (right panel) mice. Volcano scatter plots show –log_10_P_adj_ and log_2_FC. Red and green colors indicate up- and down-regulated DEGs, respectively. Thresholds (log_2_FC > 1 or log_2_FC < –1) and –log_10_P_adj_ < 0.1 are indicated by yellow dashed lines. Selected DEG symbols are shown. **(C)** Principal component analysis of female (red) and male (blue) groups of sham (rectangles) and axotomy (circles) samples (*n* = 3 samples/group). Red and blue arrows indicate genes with the most influence on variance in females and males, respectively. **(D)** A principal component analysis (PCA) loading plot indicates genes influencing variance in the top five PCA projections (PC1 to PC5) within the top/bottom 5% of the loadings range. The dot color scale corresponds to component loading coefficients defined as the coordinates of the variables divided by the square root of the eigenvalue associated with the component.

Heatmaps (Figure 2A) and volcano plots (Figure 2B) present the identity of the most significant protein-coding DEGs in each sex. Principal component analysis (PCA) identified their contribution to variance in axotomized samples from males and females based on normalized count ranking. Sample-to-sample comparisons using PCA demonstrated that female and male samples aggregated as distinct clusters in the PC1/PC2 dimensions (Figure 2C).

The most significant DEGs that contributed to gene expression variance in females encoded a major acute-phase serum amyloid A-1 protein (*Saa1*), leucine-rich repeat-containing protein 15 (*Lrcc15*), neuronal pentraxin-2 (*Nptx2*), heterodimeric neurotropic cytokine receptor-like factor 1 (*Crlf1*), cardiotrophin-like cytokine factor 1 (*Clcf1*), and sonic hedgehog protein (*Shh*). In males, DEGs encoding the potassium channel subfamily T member 1 (*Kcnt1*), apoptosis-promoting dendrin (*Ddn*), inactive serine/threonine-protein kinase (*Plk5*), Toll-like receptor 9, and myelin-associated oligodendrocyte basic protein (*Mobp*) were the most significant drivers of variance. These and other DEGs identified by PCA (Figures 2C,D) could present potentially important sex-specific markers of early PNS injury response and nerve regeneration.

### Gene ontology analysis identified sexual dimorphic genes relevant to peripheral nervous system (PNS) regeneration

**FIGURE 3:**
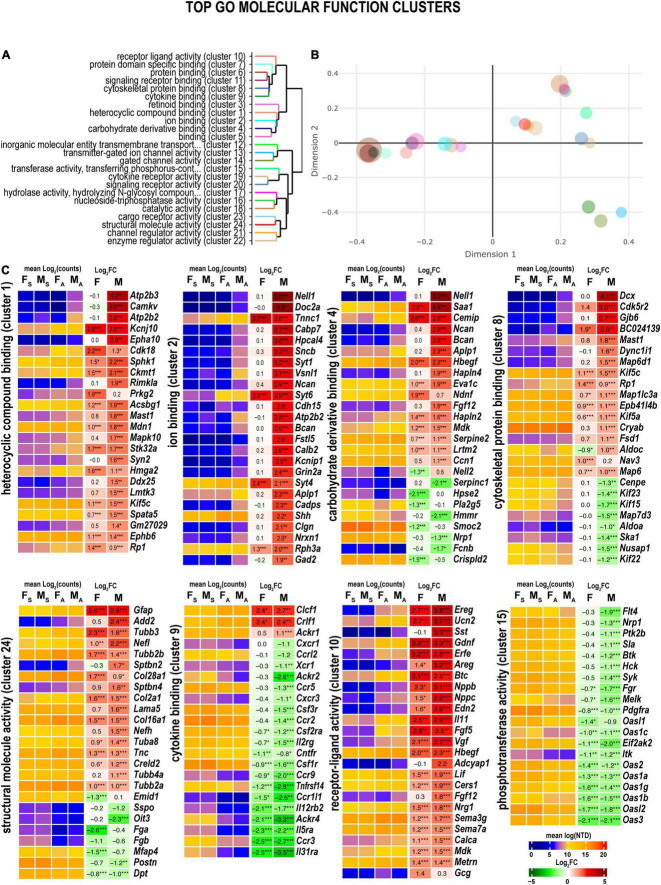
Axotomy influenced distinct molecular functions in each sex. **(A)** Clustering of significant gene ontology (GO) molecular functions based on best-match average (BMA) distances. **(B)** Wang distances of GO molecular function clusters projected on a two-dimensional scaling plot using the *ViSEAGO* R package. **(C)** Heatmaps of differentially expressed genes (DEGs) associated with eight GO molecular functions: heterocyclic compound binding (cluster 1), ion binding (cluster 2), carbohydrate derivative binding (cluster 4), cytoskeletal protein binding (cluster 8), cytokine binding (cluster 9), receptor-ligand activity (cluster 10), phosphotransferase activity (cluster 15), and structural molecule activity (cluster 24). Heatmaps display normalized mean log_2_(counts) in sample groups (F_S_, female sham; M_S_, male sham; F_A_, female axotomy; M_A_, male axotomy) and log_2_FC in female (F) and male (M); (*n* = 6 mice/group, 2 mice/sample (pooled), 3 sample/group) of respective DEGs. Log_2_FC significance was determined in DESeq2 using Wald test: *, *p* ≤ 0.05; **, *p* ≤ 0.005; ***, *p* ≤ 0.0005. DEGs were sorted by log_2_FC.

Many sexually dimorphic and monomorphic DEGs were recognized as important components of cell signaling pathways in the nervous and immune systems. To gain further mechanistic insight into their role in nerve regeneration, gene ontology (GO) enrichment analysis was conducted. Molecular-level biochemical characteristics of DEGs were used to identify the most relevant GO clusters of molecular function (Figures 3A,B). GO clusters that included sexually dimorphic DEGs relevant to nerve injury response are illustrated in Figure 3C and detailed below.

#### Heterocyclic compound binding and ion binding proteins

Heterocyclic compound binding proteins interact with ATP, GTP, nucleobases, and their derivatives (cluster 1). Subunits of the ATP-driven Ca^2+^ ion pump (*Atp2b2, Atp2b3*), CaM kinase-like vesicle-associated protein (*Camkv*), ephrin type-A receptor 10 (*Epha10*), and N-acetylaspartylglutamate synthase A (*Rimkla*) were significantly upregulated in male nerves. In females, cyclin-dependent kinase 18 (*Cdk18*) and cGMP-dependent protein kinase 2 (*Prkg2*) were upregulated. Notably, most proteins that bind charged ions (cluster 2) were upregulated only in males, including the protein kinase C-binding protein Nell1, and Ca^2+^ sensors regulating vesicular release, the double C2-like domain-containing protein α (*Doc2a*), and Ca^2+^-binding protein 7 (*Cabp7*), the hippocalcin-like protein 4 (*Hpcal4*), and β-synuclein (*Sncb*) involved in neuronal plasticity.

#### Carbohydrate derivative binding

Several carbohydrate-binding proteins (cluster 4) were downregulated in female nerves, including the protein kinase C (PKC)-binding protein (*Nell2*), inactive heparanase-2 (*Hpse2*), phospholipase A2 (*Pla2g5*), matrix-assembly related SPARC-related modular Ca^2+^-binding protein (*Smoc2*), and a cysteine-rich secretory protein LCCL (*Crispld2*). In males, these proteins exhibited high levels of expression. In addition, genes encoding Nell1, neuronal adhesion neurocan core protein (*Ncan*), brevican core protein (*Bcan*), and β-amyloid precursor protein (*Aplp1*) were upregulated and expressed in males.

#### Cytoskeletal binding and structural proteins

Male nerves displayed higher up-regulation of the microtubule-associated neuronal migration protein doublecortin (*Dcx*), the actinin-interacting Cdk5 activator Cdk5r2, Gap junction β6 protein (*Gjb6*), GAR domain-containing protein (*BC024139*), the microtubule-stabilizing MAP6 domain-containing protein (*Map6d1*), cytoplasmic dynein intermediate (*Dync1i1*), and the microtubule-associated serine/threonine-protein kinase (*Mast1*) (cluster 8). Oppositely, mRNAs encoding kinesin-like motor enzymes Kif15/Kif22/Kif23, nucleolar and spindle associated protein (*Nusap1*), spindle/kinetochore-associated protein (*Ska1*), and a centromere-associated protein (*Cenpe*) were reduced in males. Microtubule-associated oxygen-regulated protein Rp1 and neuron navigator Nav3 demonstrated more robust upregulation in female nerves.

Genes encoding structural proteins demonstrated strong upregulation in both sexes (cluster cl24) of tubulin (*Tubb3, Tubb2b, Tuba8*, and *Tubb2a*), collagen (*Col28a1*, *Col2a1*, and *Col16a1*), and glial fibrillary acidic protein (*Gfap*) genes. In contrast, β-adducin (*Add2*), light and heavy chains of neurofilament (*Nefl* and *Nefh*, respectively), spectrin β chain (*Sptbn2*), and Laminin subunit α5 (*Lama5*) showed male-dominant upregulation.

#### Cytokine binding

Reduced expression of many cytokine receptors was observed in both sexes (cluster 9), including interleukin receptors (*Il31ra, Il5ra, Il12rb2*), chemokine receptors (*Ccrl2, Ccr5, Ccr2, Ccr9, Ccr1l1*, and *Ccr3*), and other receptors (*Tnfrsf14, Csf1r, Ackr1*, and *Ackr4*). Despite the decrease, the absolute expression levels of many cytokine receptor genes remained high. Only cytokine receptor-like factor 1 (*Crlf1*) and corticotrophin-like cytokine factor 1 (*Clcf1*) demonstrated significant upregulation in both sexes.

#### Receptor ligand activity

Both males and females exhibited significant upregulation of ligand-encoding DEGs (cluster 10), including urocortin 2 (*Ucn*2), nerve growth factor inducible (*Vgf*), endothelin (*Edn2*), calcitonin (*Calca*), glial cell differentiation regulator meteorin (*Metrn*), leukemia inhibitory factor (*Lif*), glial-derived neurotrophic factor (*Gdnf*), epidermal growth factor β-cellulin (*Btc*), epidermal growth factor-like (*Egfl8*), and fibroblast growth factor (*Fgf5*). Male-specific upregulation of somatostatin (*Sst*), pituitary adenylate cyclase-activating polypeptide (*Adciap1*), cholecystokinin (*Cck*), neuropeptide galanin (*Gal*), ghrelin (*Ghrl*), and pro-opiomelanocortin (*Pomc*). Proglucagon (*Gcg*) and stanniocalcin-1 (*Stc1*) exhibited upregulation in females.

#### Phosphotransferase activity

Phosphotransferases (cluster 15), including non-receptor tyrosine kinases encoded by the *Btk, Hck, Syk*, and *Itk* genes, decreased in male nerves. In addition, both female and male nerves showed reduced expression of multiple members of the interferon-induced 2’-5’-oligoadenylate synthases members (*Oas3, Oasl2, Oas1b, Oas1g, Oas1a, Oas2, Oas1c*, and *Oasl1*).

### Axotomy significantly reshaped cell signaling in regenerating nerves

Signaling pathways, upstream regulation, and interactive networks were predicted by Ingenuity Pathway Analysis (IPA) software using a highly stringent subset of DEGs (P_adj_ < 0.001 and log_2_FC > 1 or log_2_FC < –1). Significant canonical pathways were further ranked based on Fisher’s exact test (*p* < 0.05). Positive or negative z-scores determined pathways’ activation and deactivation.

**FIGURE 4:**
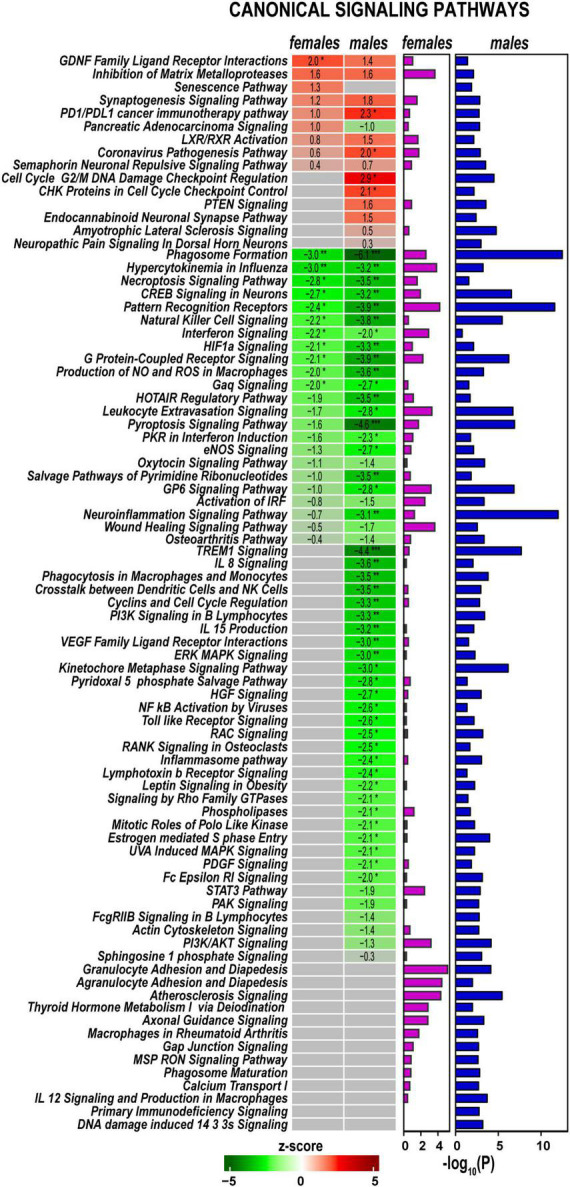
Canonical signaling pathways demonstrate distinct regulation patterns in females and males. Canonical pathways were predicted in Ingenuity Pathway Analysis (IPA) and ranked by z-scores. Positive (red) and negative (green) z-scores indicate pathway up-regulation or down-regulation, respectively, according to the color scale. Fisher’s exact test *p*-values [–log_10_(P)] predicted for each canonical pathway are shown by purple (females) and blue (males) horizontal bars. Asterisks indicate significance *, z > 2.0 or z <−2; **, z > 3.0 or z <−3; ***, z > 4.0 or z <−4.

Axotomy-induced significant changes in cellular signaling processes in both sexes (Figure 4), including pathways with female- or male-specificity. Accordingly, *GDNF Family Ligand Receptor Interactions*, *Inhibition of Matrix Metalloproteinases, Synaptogenesis Signaling Pathway, LXR/RXR Activation, PD1/PDL1 cancer immunotherapy pathway*, and *Coronavirus Pathogenesis Pathway* demonstrated activation in both sexes. *PTEN Signaling, CHK Proteins in Cell Cycle Checkpoint Control, Cell Cycle G2/M DNA Damage Checkpoint Regulation*, and *Endocannabinoid Neuronal Synapse* pathways were activated in males. In females, *Senescence Pathway* and *Pancreatic Adenocarcinoma Signaling* were activated. Many pathways exhibited downregulation in both sexes, including *Neuroinflammation Signaling, Pattern Recognition Receptors, Phagosome Formation, Pyroptosis Signaling, Leukocyte Extravasation Signaling, HOTAIR Regulatory Pathway, G Protein-Coupled Receptor Signaling*, and *Natural Killer Cell Signaling*. In addition, males specifically reduced *PI3K/AKT Signaling, Kinetochore Metaphase Signaling, IL-8 Signaling*, and *TREM1 Signaling* pathways. In both sexes, regenerating nerves displayed activation of *Macrophages in Rheumatoid Arthritis, Axonal Guidance, Thyroid Hormone Metabolism I via Deiodination, Atherosclerosis Signaling, and Agranulocyte/Granulocyte Adhesion and Diapedesis* canonical pathways.

### Upstream regulator molecules

**FIGURE 5:**
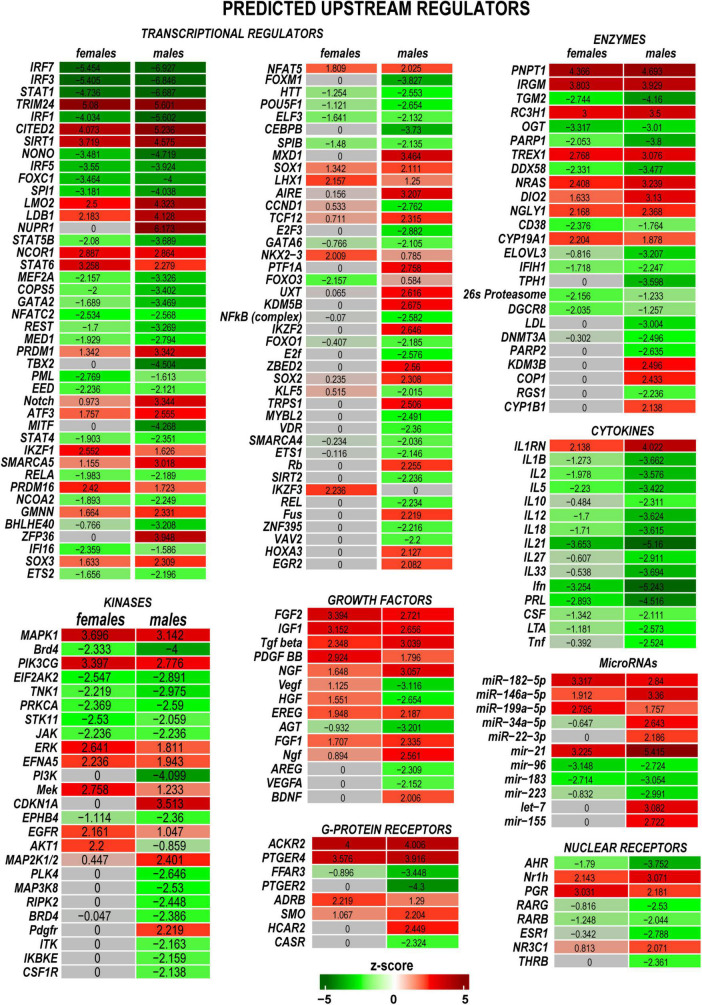
Upstream regulators of the post-injury response included transcriptional regulators, kinases, growth factors, G-protein receptors, enzymes, cytokines, microRNAs, and nuclear receptors. Positive (red) and negative (green) z-scores indicate regulator effects to upregulate or downregulate respective differentially expressed genes (DEGs). The color scale represents the z-score range.

Predictive analysis of upstream regulators was conducted in IPA to identify regulatory molecules, including transcription factors (TFs), any gene or small molecule that, with high probability, could affect the expression of their target DEGs. The activation or inhibition efficiency of each upstream regulator on target DEGs could be defined by positive or negative z-scores, respectively, as illustrated in Figure 5 and summarized in Supplementary Table 2. Worth noting that upstream regulator z-scores do not directly reflect the expression of the regulator itself (Krämer et al., 2014), as different organs or tissues can contribute regulatory molecules. IPA identified many sexually dimorphic upstream regulators, including the brain-derived neurotrophic factor (Bdnf), a known stimulator of the nerve axon growth (Zhou and Shine, 2003). Bdnf was predicted to strongly affect gene expression in males but not females. Bdnf level was low in the proximal nerve stumps in both sexes. However, Bdnf mRNA was highly abundant in DRG and demonstrated a male-dominant increase post-axotomy (Chernov and Shubayev, 2021). Other male-specific upstream positive regulators included Sox2, Aire, Egr2, Cdkn1a, Map2k1/2, and other molecules. Ikzf3, Mek, Erk, and Akt1 were predicted as prospective positive regulators in females. Remarkably, IRFs, STATs, and cytokines were identified among significant negative regulators in both sexes.

### Sexually dimorphic protein phosphorylation

**FIGURE 6:**
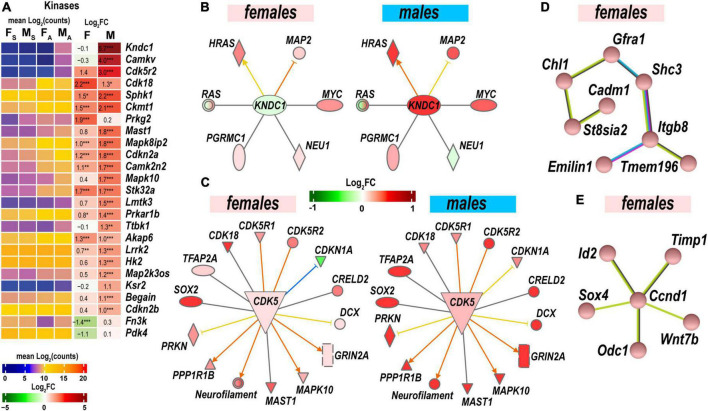
Protein phosphorylation was strongly activated to regulate cell-cell adhesion and neuronal plasticity. **(A)** Expression of differentially expressed genes (DEGs) with kinase activities. Heatmaps display normalized mean log_2_(counts) in sample groups (F_S_, female sham; M_S_, male sham; F_A_, female axotomy; M_A_, male axotomy) and log_2_FC in female (F) and male (M); (*n* = 6 mice/group, 2 mice/sample (pooled), 3 sample/group) of respective DEGs. Log_2_FC significance was determine in DESeq2 using Wald test: *, *p* ≤ 0.05; **, *p* ≤ 0.005; ***, *p* ≤ 0.0005. DEGs were sorted by log_2_FC. **(B)** RAS-Guanine nucleotide exchange factor KNDC1 links HRAS, MYC, NEU1, RAS, and MAP2. Male but not female nerves exhibit upregulation of key components. **(C)** Protein interaction networks regulated by CDK5 in females (left) and males (right). **(D)** Female-specific Gfra1/Cadm1 axis. **(E)** Proposed regulation by female-prevalent Ccnd1/Timp1 axis. The shape’s colors on panels B and C correspond to up- (red) or down- (green) regulation of respective DEGs according to the Log_2_FC scale. Protein interactive networks were predicted in IPA (panels **B** and **C**) and the STRING database (panels **D** and **E**).

Protein kinases and their regulatory molecules were identified as significant sexually dimorphic upstream regulators (Figure 6A and Supplementary Table 2). Male-specific upregulation of Kndc1 (also known as Very-KIND) may modulate Hras, Myc, Neu1, Ras, and Map2 (Figure 6B) during neuronal growth (Huang et al., 2007). Highly expressed Cdk5 was moderately activated by axotomy in male nerves. Cdk5 activator molecule that also controls cytoplasmic or nuclear localization of the Cdk5 complex demonstrated male-dominant upregulation. Co-upregulation of Cdk5-interacting and target molecules in males was significantly stronger than in females (Figure 6C). While female nerves upregulated fewer protein-coding DEGs than males, two female-specific interactive networks were determined using the STRING database (Szklarczyk et al., 2021). The first proposed network potentially conveys Gdnf signaling *via* the neuron-specific adaptor Shc3, the Gdnf receptor α1 (Gfra1), and neural cell adhesion proteins Chl1 and Cadm1 to regulate cell-cell adhesion and neuronal plasticity (Figure 6D). Another network is centered around the regulatory component of the Cyclin D1 (Ccnd1)-Cdk4 complex (Figure 6E). It is known to respond to Timp1 that activates cyclic AMP-induced regenerative program gene post-axotomy (Liu et al., 2015) partly *via* Cd63/Pi3k/Akt signaling (Rossi et al., 2015).

### Cell signaling engages sexually dimorphic molecular programs

Regulatory signaling events reflect distinct molecular programs involved in regenerating nerves. Programs exhibiting sexually dimorphic regulation are as follows:

#### Neuronal survival and regeneration programs

**FIGURE 7:**
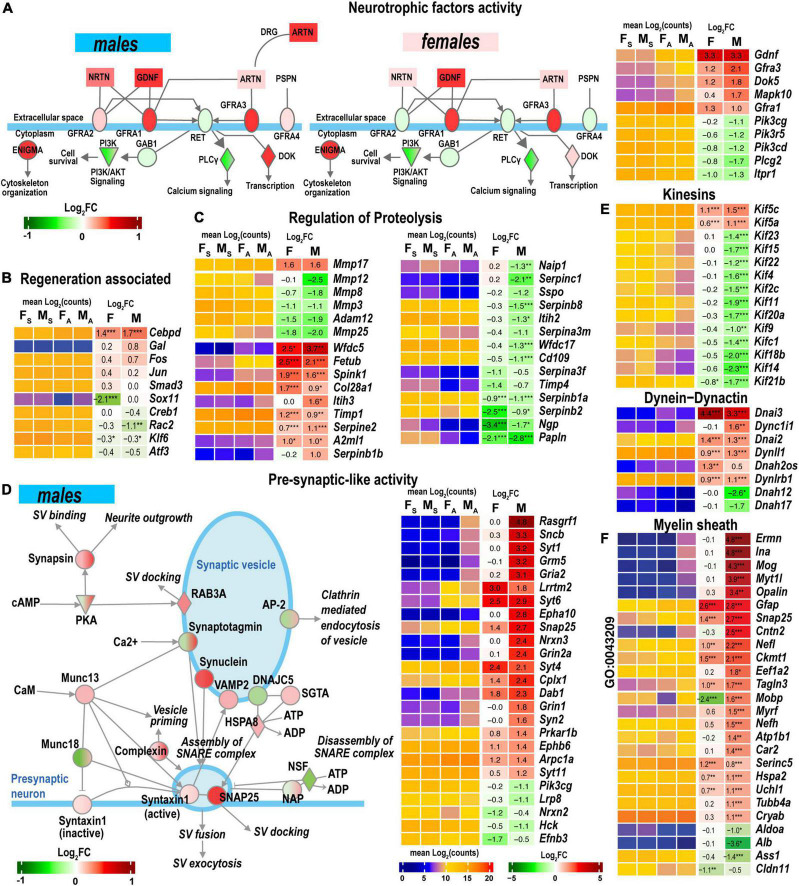
Nerve regeneration-related signaling and pre-synaptic-like activity exhibited sexually dimorphic activation. **(A)** glial cell line-derived neurotrophic factor (GDNF) family of ligands can drive the cytoskeleton organization, Pi3k/Akt signaling, Ca^2+^-signaling, and regulate downstream gene transcription. The diagram partially corresponds to the *GDNF Family Ligand Receptor Interactions Pathway* predicted by Ingenuity Pathway Analysis (IPA). Colors correspond to up- (red) or down- (green) regulation of respective differentially expressed genes (DEGs) according to the Log_2_FC scale. Protein interactive networks were predicted in IPA. **(B)** Regeneration-associated DEGs. **(C)** Regulation of extracellular proteolysis-related DEGs. **(D)** Pre-synaptic-like activity in male nerves. The diagram partially corresponds to the *Synaptogenesis Signaling Pathway* predicted by IPA. Colors correspond to up- (red) or down- (green) regulation of respective DEGs according to the Log_2_FC scale. **(E)** Kinesins, dynein-dynactin DEGs. F, Myelin sheath-related DEGs. Heatmaps on panels **A–C, E**, and **F** display normalized mean log_2_(counts) in sample groups (F_S_, female sham; M_S_, male sham; F_A_, female axotomy; M_A_, male axotomy) and log_2_FC in female (F) and male (M); (*n* = 6 mice/group, 2 mice/sample (pooled), 3 sample/group) of respective DEGs. Log_2_FC significance was determined in DESeq2 using Wald test: *, *p* ≤ 0.05; **, *p* ≤ 0.005; ***, *p* ≤ 0.0005. DEGs were sorted by log_2_FC.

*GDNF Family Ligand Receptor Interactions* pathway is modulated by the Gdnf and related ligands: neurturin (*Nrtn*), artemin (*Artn*), and persephin (*Pspn*). Notably, the *Gdnf* gene was highly upregulated in both sexes in the proximal stumps (Figure 7A) but not in DRG (Chernov and Shubayev, 2021). Neurturin was upregulated in males. Artemin was mildly expressed in distal stumps but significantly upregulated in male DRG. The ligand receptors encoded by Gfra1 and Gfra3 genes increased in both sexes.

Regeneration-associated genes (RAGs) were rapidly induced by axotomy in the corresponding DRG (Chernov and Shubayev, 2021). *Fos, Jun, Smad3, Creb1, Rac2, Klf6*, and *Atf3* mRNAs were detected in the nerves (Figure 7B). Cebpd, a bZip-containing CCAAT/enhancer-binding protein δ, increased in both sexes. The *Sox11* gene was 4-fold explicitly downregulated in females.

#### Extracellular matrix (ECM) homeostasis and regulation of proteolysis

Both male and female nerves upregulated matrix metalloproteinase (MMP) Mmp17 (MT4-MMP) expression (Figure 7C). Mmp24 moderately increased only in males. A group of MMP genes co-localized in the 9qA1 locus of the mouse chromosome 9, Mmp8, Mmp12, Mmp13, and Mmp27, synchronously decreased only in males. Mmp25 decreased in both sexes. Other MMP genes, including Mmp2, Mmp3, Mmp14, Mmp15, and Mmp19, demonstrated high expression levels in both sham and axotomy nerves irrespective of sex.

Many endogenous inhibitors of proteinases exhibited sexually dimorphic regulation of gene expression, including the WAP domain proteinase inhibitor (*Wfdc5*), the cysteine protease inhibitor (*Fetub*), and *s*erine protease inhibitor Kazal-type 1 (*Spink1*). *Timp1* increased in both sexes, in agreement with our previous reports (Kim et al., 2012; Liu et al., 2015; Chernov and Shubayev, 2021). The glia-derived nexin (Serpine2) was upregulated, but other serine protease inhibitors of the serpin family were downregulated. Inter-α-trypsin inhibitor heavy chain H3 (Itih3) and serine proteinase inhibitor Serpinb1b demonstrated male-specific upregulation.

#### Regulation of synaptogenesis signaling

Synaptogenesis Signaling Pathway was distinctly regulated in both sexes. Genes encoding components of synaptic vesicles (SV) showed greater activation in males (Figure 7D). Consequently, the male-dominant increase of the pre-synaptic-like activity and vesicular transport can be mediated by synuclein subunits α, β, and γ, Rab3A oncogene, and vesicle-associated membrane protein (*Vamp2*). It is important to note that SV-related genes show high levels of expression in sham and axotomized female nerves; therefore, male-prevalent gene activation could level up pre-synaptic activity to match the activity in females.

#### Axonal transport

Axonal transport is essential for neuronal survival and function restoration after damage. Many kinesins involved in anterograde axonal transport decreased in males (Figure 7E), except Kif5 family members increased in males. Retrograde transport-related axonemal dynein/dynactin encoding genes showed remarkable sexually dimorphic regulation. Most notably, dynein intermediate chain mRNAs Dnai2 and Dnai3 highly increased in both sexes. Dynein heavy chain mRNA Dnah12/17 decreased in males.

#### Myelin sheath

Male nerves upregulated myelinogenesis- and neuron morphogenesis-related DEGs (according to GO term GO:0043209), including ermin (*Ermn*), α-internexin (*Ina*), myelin oligodendrocyte glycoprotein (*Mog*), opalin (*Opalin*), contactin 2 (*Cntn2*) (Figure 7F). The expression of these DEGs in females remained low. Other myelin sheath-related molecules were represented in both sexes and exhibited further upregulation in males. Notably, myelin-associated oligodendrocyte basic protein (*Mobp*) demonstrated significant sexually dimorphic regulation by upregulating in males and downregulating in females.

#### Regulation of neuroreceptors, transporters, and ion channels

**FIGURE 8:**
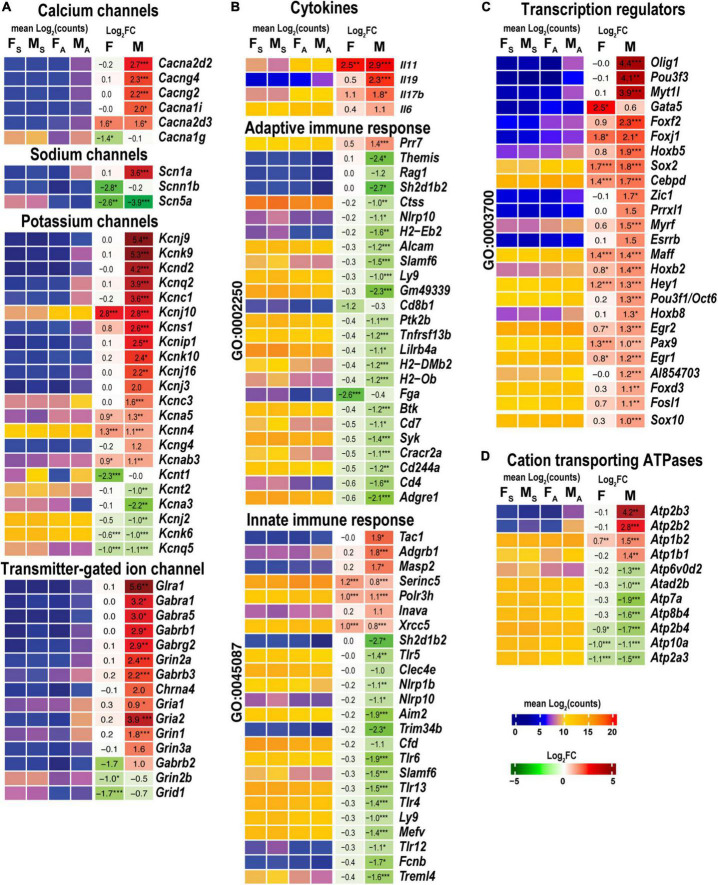
Males exhibited stronger activation of ion channels, transcriptional regulators, cation transporting ATPases, and select cytokines. Innate and adaptive immunity exhibited a male-specific decrease. **(A)** Ion channel genes. **(B)** Cytokines, adaptive (Go term GO:0002250), and innate (GO term GO:0045087) immunity-related DEGs. **(C)** DNA-binding transcriptional regulators (GO term GO:0003700). **(D)** Cation transporting ATPases. Heatmaps display normalized mean log_2_(counts) in sample groups (F_S_, female sham; M_S_, male sham; F_A_, female axotomy; M_A_, male axotomy) and log_2_FC in female (F) and male (M); (*n* = 6 mice/group, 2 mice/sample (pooled), 3 sample/group) of respective DEGs. Heatmap colors correspond to the respective scales. Log_2_FC significance was determined in DESeq2 using Wald test: *, *p* ≤ 0.05; **, *p* ≤ 0.005; ***, *p* ≤ 0.0005. DEGs were sorted by log_2_FC.

Male, but not female, nerves demonstrated increased levels of transmitter-gated ion channel mRNAs (Figure 8A) encoding ionotropic γ-aminobutyric acid (GABA) receptors (*Gabra1, Gabra5, gabrg2, Gabrb1, Gabrb2, Gabrb3*, and *Gabrg1*), members of ionotropic glutamate receptor N-methyl-D-aspartic acid (NMDA) receptor subunits (*Grin1, Grin2a*, and *Grin3a*), α-amino-3-hydroxy-5-methyl-4-isoxazole propionic acid (AMPA) receptor subunits Gria1 and Gria2, kainic acid receptor subunit Grik1. Notably, mRNAs encoding 5-hydroxytryptamine 2C (serotonin) receptors Htr2c and Htr3a also increased. Male nerves exhibited increased potassium, calcium, and sodium voltage-gated ion channel-related mRNAs. Remarkably, levels of these mRNAs diminished in the respective DRGs measured in the same animal cohorts (Chernov and Shubayev, 2021), suggesting the involvement of sex-specific axonal mRNA transport in the redistribution of injury-related mRNAs during early-phase response. Female nerves showed no change in ion-channel mRNA levels, except for *Cacna1g, Scb5a, Kcnt1*, and *Kcnq5, demonstrating* a reduction.

#### Immune regulation

Cytokines Il11 and Il17b were upregulated in both sexes, while Il6 and Il19 were predominantly activated in males (Figure 8B). Genes related to adaptive (GO:0002250) and innate (GO:0045087) immunity showed a system-wide male-specific decrease. Many immunity-related canonical pathways were significantly downregulated after axotomy in both sexes’ proximal nerve segments, including *Neuroinflammation Signaling Pathway, Activation of IRF, Leukocyte Extravasation, Signaling, Interferon Signaling, Pattern Recognition Receptors*, and *Phagosome Formation* pathways (Figure 4). The following immunity-related pathways demonstrated male-specific negative regulation: *Inflammasome Pathway, Toll-like Receptor Signaling, IL-15 Production, PI3K Signaling in B Lymphocytes, Phagocytosis in Macrophages* and *Monocytes, and IL-8 Signaling.*

#### Transcription regulators

Male nerves significantly upregulated neuronal differentiation and reprogramming-related TFs, the myelin transcription factor 1-like protein (Myt1l), and POU domain-containing Pou3f3 (Figure 8C). Upregulation of oligodendrocyte transcription factors 1/2 (*Olig1/2*), neurogenesis-related TFs (*Zic1, Pou3f1/Oct6, Esrrb, Myrf*, and *Prrxl1*), and nuclear protein Nupr1 were male-specific. Also, males upregulated Fox-family TFs (*Foxf2, Foxj1, Foxd3*, and *Foxc2*), Hox-family TFs (*Hoxb5, Hoxb2, Hoxb8*, and *Hoxb3os*), and Sox-family TF genes (*Sox2* and *Sox10*), and the RNA-binding proteins ZFP36 known to restrain T cell activation and antiviral immunity (Moore et al., 2018). Sox2 and Sox4 TFs increased in females, but Sox11 decreased after axotomy. mRNA level of Gata5 was higher in females than in males. We concluded that post-axotomy regulation of TFs is characterized by sexually dimorphic patterns to potentially execute distinct regenerative programs in the PNS of females and males.

#### Cation transporting ATPases

Males but not females upregulated plasma membrane Ca^2+^-transporting ATPase subunits Atp2b2 and Atp2b3 (Figure 8D). mRNAs of Na^+^/K^+^-transporting ATPase subunits Atp1b1 and Atp1b2 were abundant in both sexes and exhibited further increase in males.

#### Non-coding RNAs

**FIGURE 9:**
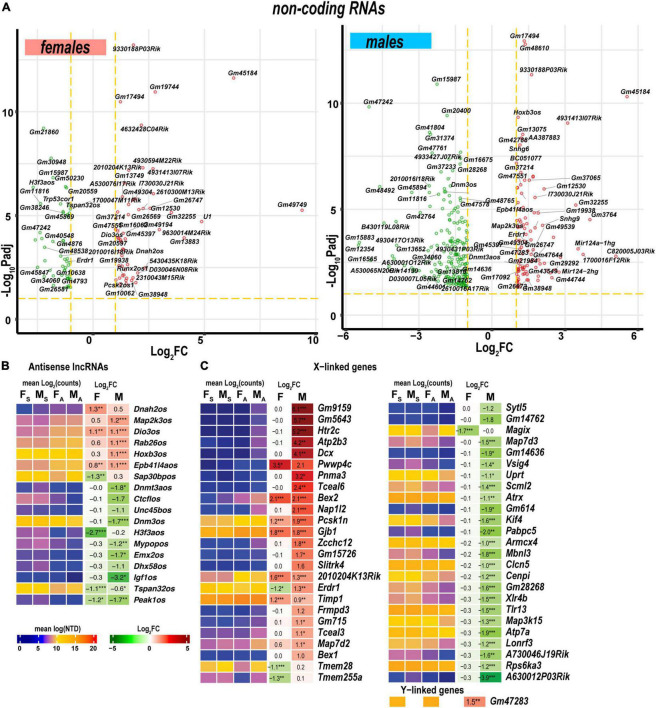
Regulated ncRNA and sex chromosome-linked differentially expressed genes (DEGs) exhibited profound sexual dimorphism. **(A)** Significant ncRNA DEGs in female (left panel) and male (right panel) mice. Volcano scatter plots show –log_10_P_adj_ and log_2_FC. Red and green colors indicate up-and downregulated ncRNAs, respectively. Thresholds (log_2_FC > 1 or log_2_FC < –1) and –log_10_P_adj_ < 0.1 are shown by yellow dotted lines. Selected DEGs are labeled by gene symbols. **(B)** Regulated antisense lncRNA DEGs. **(C)** Regulated sex chromosome-linked DEGs. Heatmaps display normalized mean log_2_(counts) in sample groups (F_S_, female sham; M_S_, male sham; F_A_, female axotomy; M_A_, male axotomy) and log_2_FC in female (F) and male (M); (*n* = 6 mice/group, 2 mice/sample (pooled), 3 sample/group) of respective DEGs. Heatmap colors correspond to the respective scales. Log_2_FC significance was determined in DESeq2 using Wald test: *, *p* ≤ 0.05; **, *p* ≤ 0.005; ***, *p* ≤ 0.0005. DEGs were sorted by log_2_FC.

Levels of *Mir124-1hg* and *Mir124-hg2* RNAs, the precursors of the ubiquitous neuronal microRNA Mir-124, were higher in males than in females (Figure 9A). In addition, small nucleolar RNAs (snoRNAs) encoded by *Snhg9, Snhg6*, and *Snora57* host genes showed male-dominant expression. The erythroid differentiation regulator 1 ncRNA encoded by the X-linked Erdr1 gene was upregulated in males but downregulated in females. U1 spliceosome RNA (U1) and ribonuclease P RNA component H1 (Rpph1) were upregulated in females.

Antisense lncRNAs (asRNA) can potentially target co-localized PNS injury-related genes *via* cis-acting mechanisms. In females, genes encoding the axonemal dynein (*Dnah2*), tetraspanin 32 (*Tspan32*), pseudopodium-enriched atypical kinase 1 (*Peak1*), SAP30 binding protein (*Sap30bp*), and H3.3 histone (*H3f3a*) could be targeted by the respective asRNA (Figure 9B). In males, asRNAs could target genes encoding deiodinase (*Dios3*), MAP2 kinase (*Map2k3*), RAB26 oncogene (*Rab26*), homeobox B2/B3 (*Hoxb2, Hoxb3*), dynamin 3 (*Dnm3*), the transcriptional repressor CCCTC-binding factor (*Ctcf*), *de novo* DNA methyltransferase 3a (*Dnmt3a*), and insulin-like growth factor 1 (*Igf1*).

#### Sex chromosomes

As reported in the DRGs of the same animal cohort (Chernov and Shubayev, 2021), genomic localization of DEGs on a sex-chromosome could potentially determine sexually dimorphic expression. In the proximal nerve stumps, most X-linked DEGs exhibited male-specific regulation (Figure 9C). However, several female X-linked DEGs, including *Magix, Tmem255a, Tmem28, Map7d2, Timp1, Erdr1, 2010204K13Rik, Gjb1, Pcsk1n, Bex2*, and *Pwwp4c*, were elevated in both sexes. Male-specific Y-linked transcript Gm47283 was upregulated post-axotomy in males.

## Discussion

**FIGURE 10:**
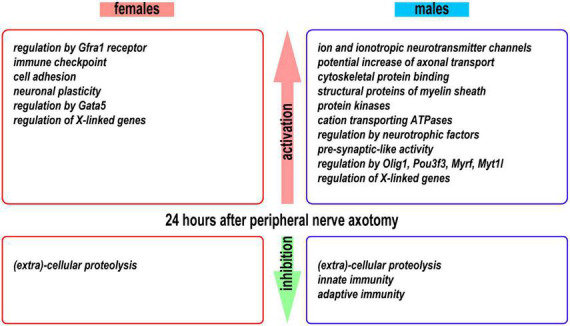
A summary of activated and inhibited transcriptional programs detected in the proximal nerve stumps 24 h post-axotomy in female and male mice.

We obtained novel evidence of sexually monomorphic and dimorphic regulation of the early-phase transcriptional programs in regenerating segments of injured peripheral nerves (Figure 10). The concerted accumulation of mRNAs that encode actin, microtubules, neurofilaments, cytoskeletal regulators, and cytoskeleton-binding proteins was observed in proximal nerve stumps of both sexes, confirming extensive cytoskeletal remodeling aimed at rebuilding the growth cone (Lundborg, 1987; Burnett and Zager, 2004; Radtke and Vogt, 2009).

### Schwann cell reprogramming

After nerve injury, Schwann cells assume a pro-regenerative function (Jessen and Mirsky, 2016, 2021; Merrell and Stanger, 2016; Milichko and Dyachuk, 2020), exhibiting an exceptional phenotypic plasticity (Jessen and Arthur-Farraj, 2019; Stierli et al., 2019; Nocera and Jacob, 2020) that supports their de-differentiation, proliferation, and re-differentiation into myelinating and non-myelinating phenotype to facilitate repair of the respective axons. Schwann cell reprogramming is controlled by a set of TFs (Balakrishnan et al., 2021), including Myt1l, Pou3f1/Oct6, Myrf, Olig1/2, Jun, Sox-, Hox-, and Fox-family members TFs upregulated at 24 h post-axotomy predominantly in males. Transcripts of TF genes, such as *Fos, Smad3, Creb1, Rac2, Klf6*, and *Atf3*, that function as RAGs (reviewed in [Van Kesteren et al., 2011; Ma and Willis, 2015)] in the corresponding DRGs after PNS injury (Chernov and Shubayev, 2021) were detected in proximal nerve segments. In addition to transcriptional activity in cells of the PNS, this accumulation may reflect an anterograde transport from DRG in coordinated axonal regeneration processes post-axotomy. Single-cell RNA-seq and spatial transcriptomics analysis could provide crucial information on the identity of cell lineages activating these sexually dimorphic TFs.

The GDNF family of ligands (GDNF, neurturin, artemin, and persephin), known survival factors for neurons, bind to GFRA receptors, trigger the phosphorylation of RET tyrosine kinase receptor (Durbec et al., 1996) that is known to regulate Pi3k/Akt and Plcγ/Ip3r dependent Ca^2+^ signaling during neurogenesis (Fukuda et al., 2002; Lundgren et al., 2012) and, sometimes, hyperalgesia (Bogen et al., 2008). Furthermore, GDNF/Gfra1 could interact with neural cell adhesion molecules such as NCAM to induce an axonal expansion (Nielsen et al., 2009). GDNF family of ligands can act in a synergetic manner with other growth factors, including transforming growth factor-β (Tgf-β) and sonic hedgehog (Shh) [reviewed in Jessen and Mirsky (2021)]. GDNF signaling can stimulate the migration of neuronal precursors and Schwann cells (Iwase et al., 2005) to the injury site in both sexes.

### Extracellular proteolysis

The outcome of nerve regeneration after sciatic nerve injury is partly dependent on the integrity of ECM-rich Schwann cell basal lamina and endoneurial tubes (Fawcett and Keynes, 1990). Proteolytic cleavage mediated by ECM proteinases and their endogenous inhibitors is indispensable for morphological remodeling of regenerating axons (Rivera et al., 2010; Fujioka et al., 2012; Bajor and Kaczmarek, 2013), after sciatic nerve injury (Liu et al., 2010). In addition, MMPs control the activity of critical growth factors, their ligands, and receptors in peripheral nerve, including IGF and neuregulin/ErbB (Chattopadhyay and Shubayev, 2009). Mmp17 was upregulated in both sexes and can convey inflammatory responses due to the TNFα-converting activity (English et al., 2000).

Intriguingly, a group of MMPs co-localized on the mouse chromosome 9 (Mmp3, Mmp8, Mmp12, Mmp13, and Mmp27) have decreased in both sexes. Because MMPs in the orthologous human chromosome 11q22.3 region could carry different histone modification marks (Chernov et al., 2010), epigenetic control of this set of metzincins may be involved. Consistent with our previous report, MMPs, and many disintegrin ADAM/ADAM-TS families of the ECM-remodeling metzincins demonstrate regulated expression in injured nerves (Chernov et al., 2015).

Notably, protease expression levels were high in intact and axotomized nerves. Processes of post-translational protease activation and binding to intrinsic proteinase inhibitors control potentially cytotoxic proteolytic activities. Aberrant cleavage of mediators of nociceptive signaling during neurogenesis potentially causes neuropathic pain (Remacle et al., 2015). Previously we identified Timps as key genes in the normal and damaged nerves (Kim et al., 2012; Nishihara et al., 2015) and post-axotomy DRG (Liu et al., 2015; Chernov and Shubayev, 2021). Timp1 binds and inhibits all MMPs, except Mmp14; Timp2 inhibits Mmp14 (Brew and Nagase, 2010). In the proximal nerve, Timp1 demonstrated strong upregulation in both sexes. Other upregulated endogenous inhibitors of proteinases Wfdc5, Fetuin B, Spink1, Itih3, and Serpineb1b could serve a not well-understood role in nerve damage response.

### Sex-specific control of axonal trafficking

An intact connection to DRG allows for dynamic axonal transport of protein and mRNA content between neuronal soma and the proximal segment of the axotomized nerve (Cavalli et al., 2005). The anterogradely transported mRNAs from DRG are directly used for the local protein synthesis at the site of the injury (Sahoo et al., 2018) and complemented by the transcriptional repertoire of the resident non-neuronal cells in the damaged nerve (e.g., Schwann cells, macrophages) that establish a microenvironment generally permissive for axon regeneration (Avraham et al., 2021).

Kinesins facilitate anterograde axonal transport of neurotransmitter receptors, mitochondria [reviewed in (Fan and Lai, 2021)], and also mRNAs [reviewed in (Kanai et al., 2004; Turner-Bridger et al., 2020)]. Remarkably, most kinesin mRNAs were depleted in proximal nerve segments at 24 h post-axotomy, except for members of the conventional kinesin-1 family Kif5a/b/c increased in males.

Retrograde transport of transcription factors from the site of nerve injury to DRG has been shown to facilitate the neuronal survival (Cox et al., 2008) and axon regeneration (Ito and Enomoto, 2016). In the corresponding DRGs of the same animal cohort, at 24 h post-axotomy, we observed male-specific reduction of mRNAs encoding calcium, sodium, and potassium ion channels as well as ionotropic AMPA, NMDA, and GABA receptors (Chernov and Shubayev, 2021) that correlated to their increase in proximal segments in the present study and presumably, anterograde axonal transport. We conclude that axonal transport of mRNAs contributes to sex-specific protein synthesis and downstream signaling programs both in DRG neurons and regenerating axons at the early stages of response to sensory nerve damage.

### Sex chromosome encoded genes

Sex chromosome-encoded genes were activated in regenerating sciatic nerves. Several X-linked genes were differentially regulated in male and female nerves. Among X-linked genes induced in nerves of both sexes were *Timp1*, known to promote regeneration (Kim et al., 2012; Liu et al., 2015; Nishihara et al., 2015), and *Map7d2*, involved in kinesin-mediated cargo transport into the axon (Pan et al., 2019). It is worth noting that genes associated with the female-specific epigenetic X-chromosome inactivation (Xi) process were differentially expressed in DRGs within 24 h after post-axotomy in the same animal cohort (Chernov and Shubayev, 2021).

### Conclusion

The noted remodeling represents baseline sexual dimorphism in uninjured peripheral nerves and an immediate regenerative response to nerve injury. The functional significance of the reported sexual dimorphism remains to be determined. It is conceivable that, at least partially, sex differences were equalized over the time-course of nerve injury. Single-cell RNA-seq and spatial transcriptomics analysis could provide crucial information on the identity of cell lineages activating these sexually dimorphic coding and non-coding RNAs.

## Materials and methods

### Reagents

Detailed descriptions of reagents and resources are included in Supplementary Table 3.

### Animals

Female and male C57BL6/J mice (6–8 weeks old, Jackson Labs, *n* = 24) were housed in a temperature-controlled room (22°C) on a 12 h light/dark cycle with *ad libitum* access to food and water. The mice were randomly assigned to axotomy and sham groups by sex (*n* = 6/group). Under isoflurane anesthesia, the left sciatic nerve was exposed at the mid-thigh level, followed by a complete transection using sterile microsurgery scissors. In the sham group, anatomically equivalent nerves were subjected to exposure without transection. The muscle was sutured, and the skin stapled. At 24 h after surgery, proximal segments of the sciatic nerve, corresponding to previously investigated lumbar (L)4 and L5 DRG tissues (Chernov and Shubayev, 2021), were collected for RNA isolation. All animal procedures were performed according to the Policy on Humane Care and Use of Laboratory Animals and the protocol approved by the Institutional Animal Care and Use Committee at the VA San Diego Healthcare System.

### Samples

All surgical and tissue harvesting instruments were sterilized and repeatedly treated with RNase Away reagent followed by RNase-free water rinse. Tissues were immediately submerged in 500 μl RNAlater Stabilization Solution to preserve RNA integrity, placed at 4°C overnight, then transferred for storage at –20°C. All sample groups were processed in parallel to minimize batch effects. Proximal stumps of sciatic nerve tissues were pooled from 2 mice per group for RNA purification.

### RNA purification

Nerve tissues were transferred in Trizol solution (Invitrogen) and disrupted by mechanical homogenization. Total RNAs were purified using RNeasy RNA purification reagents. RNA concentrations and quality were determined using Nanodrop absorbance ratios at 260/280 nm and 260/230 nm. RNA integrity indices were determined using the Agilent Bioanalyzer Nano RNA chip. 500 ng of total RNA samples (*n* = 6 mice/group, 2 mice/sample (pooled), 3 sample/group) with RIN ≥ 7.0 were used for RNA-seq.

### RNA sequencing (RNA-seq)

mRNA libraries were generated following the TruSeq Stranded mRNA library preparation protocol (Illumina). In brief, the Poly-A enriched mRNAs were purified using poly-T oligo coupled magnetic beads, followed by mRNA fragmentation, first and second strands synthesis, cleaning on AMPure XP magnetic beads, and 3’-adenylation. Ligation of TruSeq dual-index adapters was used for barcoding. The quality of RNA-seq libraries was validated using qPCR. Libraries were sized on Agilent Bioanalyzer DNA high sensitivity chip and normalized. RNA-seq was performed using the paired-end 100 cycle program on the NovaSeq 6000 system at the Genomics High Throughput Facility (University of California Irvine). Base calls were recorded and converted to FASTQ files containing sequencing reads and the corresponding quality scores using Illumina software. Sequencing was conducted until we acquired at least 50 million paired-end reads per sample.

### RNA sequencing (RNA-seq) data analysis

FASTQ files were uploaded to the Amazon S3 server and processed using Elastic Compute Cloud (EC2) [Amazon Web Services (AWS)] running Ubuntu Server 20.04 LTS (64-bit ARM). Data analysis steps are summarized in Supplementary Figure 1 and Supplementary Table 3. FASTQ files were filtered to remove low-quality bases, TruSeq dual-index adapter sequences, and unpaired reads using *Trimmomatic* (Bolger et al., 2014). Transcript-level quantification and mapping were performed using Salmon (Patro et al., 2017) and the Gencode M29 mouse genome. Mapping coverage was estimated in *MultiQC* (Ewels et al., 2016). Transcript- to gene-level quantifications were done using *Tximeta* (Love et al., 2020). Gene count matrices were processed in the DESeq2 (Love et al., 2014). Log_2_FC values were calculated using the Wald test and adjusted using the adaptive t-prior *apeglm* method (Love et al., 2014). Significant DEGs were identified by P_adj_ values below a false discovery rate (FDR) cutoff (P_adj_ < 0.1) (Supplementary Table 1). P_adj_ < 0.1 were used in downstream analyses unless otherwise noted. Batch effects were controlled using the *removeBatchEffect* tool (Ritchie et al., 2015) and *RUVseq* (Risso et al., 2014) functions. DEGs were visualized using *ggVennDiagramm*, *PCAtools*, *ComplexHeatmap*, and *EnhancedVolcano* R packages.

### Signaling pathway analysis

Prediction and biological interpretation of the regulated signaling pathways and upstream regulators in female and male animals was made in IPA using the default parameters (Krämer et al., 2014). Significance criteria of P_adj_ < 0.1, log_2_FC > 1 or log_2_FC < -1 were applied. Regulation directionality was estimated in IPA based on z-scores. Gene ontology terms were determined using *Geneontology*^1^ and the *STRING* database using the default parameters.

## Data availability statement

The datasets presented in this study can be found in the Gene Expression Omnibus (GEO) public repository (accession numbers: GSE182713 and GSE182709).

## Ethics statement

The animal study was reviewed and approved by Institutional Animal Care and Use Committee at the VA San Diego Healthcare System.

## Author contributions

AC: conceptualization, methodology, software, formal analysis, investigation, data curation, writing – original draft, review and editing, and visualization. VS: conceptualization, resources, project administration, funding acquisition, and writing – original draft, review and editing. All authors contributed to the article and approved the submitted version.

## Footnotes

1
http://geneontology.org

## Supplementary material

Supplementary Figure 1 RNA-seq analysis workflow.

Supplementary Table 1 DEGs in murine female and male post-axotomy (24 h) proximal nerve stumps.

Supplementary Table 2 Predicted upstream regulators.

Supplementary Table 3 Key resources description.

## Abbreviations

CNS: central nervous system
DEG: differentially expressed gene
DRG: dorsal root ganglia
FC: fold change
FDR: false discovery rate
GDNF: glial cell line-derived neurotrophic factor
IPA: Ingenuity Pathway Analysis
ncRNA: non-coding RNAs
PNS: peripheral nervous system
RNA-seq: RNA sequencing
SV: synaptic vesicle
TF: transcription factor
TLR: toll-like receptors.
