# Supplementary figures and images for: Sexually dimorphic transcriptional programs of early-phase response in regenerating peripheral nerves
Source: Front Mol Neurosci. 2022 Aug 2;15:958568. doi: 10.3389/fnmol.2022.958568 (PMC9378824; doi:10.3389/fnmol.2022.958568)

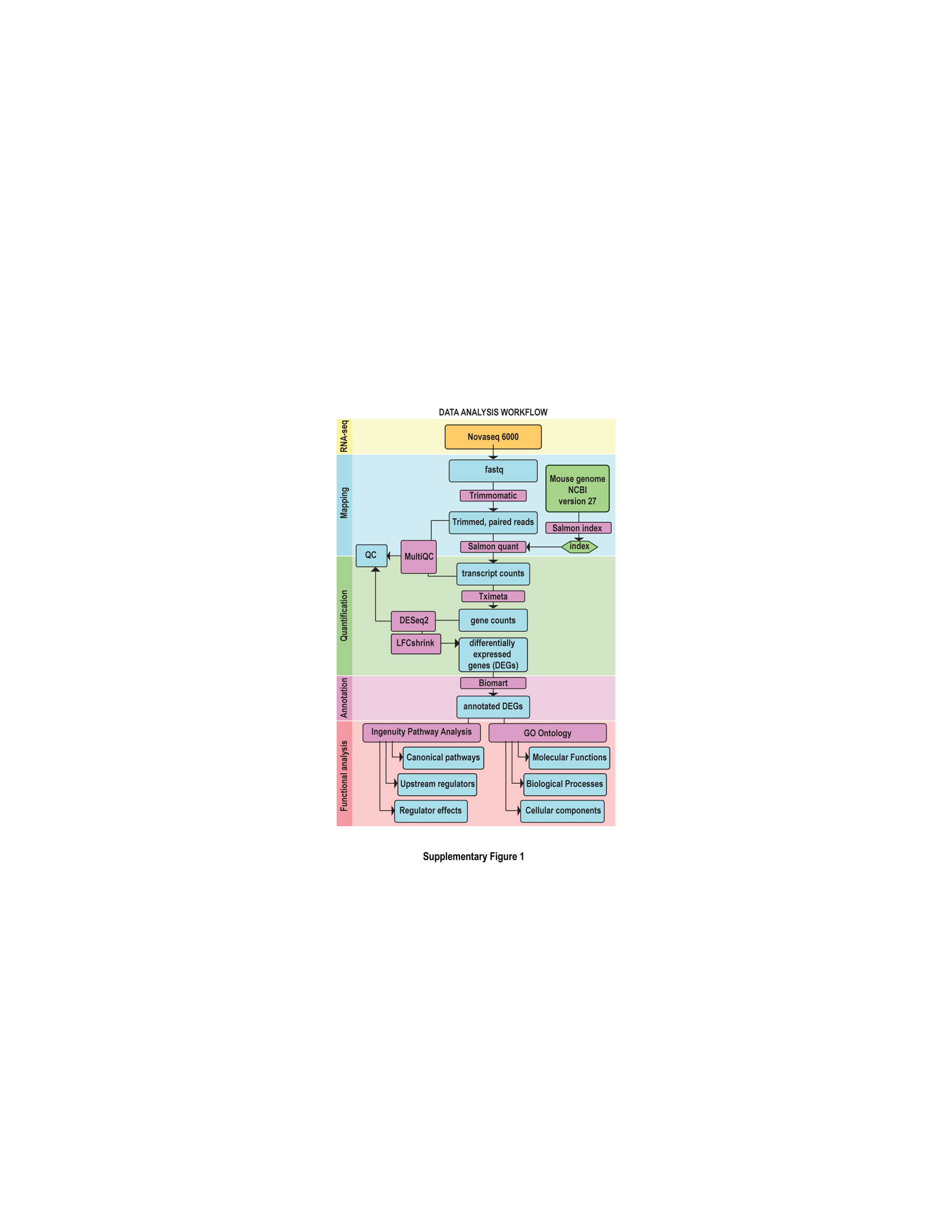

Supplement: Supplementary Figure 1 — RNA-seq analysis workflow. [file Image_1.TIF]
