# Supplementary material for: Sexually dimorphic transcriptional programs of early-phase response in regenerating peripheral nerves
Source: Front Mol Neurosci. 2022 Aug 2;15:958568. doi: 10.3389/fnmol.2022.958568 (PMC9378824; doi:10.3389/fnmol.2022.958568)
Supplement: Supplementary Table 3 — Key resources description. [file Table_3.DOCX]

**Supplementary Table 3.** Key resources description.

| Resource | Description | Source | Identifier | URL |
| --- | --- | --- | --- | --- |
| Animals | Mice | Jackson Labs, Sacramento, CA | strain C57BL/6 |  |
| Disposables | BioMasher microhomogenizer | TaKaRa | 9791B |  |
| Disposables | QIAshredder | Qiagen, Carlsbad, CA | 79656 |  |
| Reagent | Trizol reagent | Thermo Fisher Scientific, Carlsbad, CA | 15596026 |  |
| Reagent | RNAlater Stabilization Solution | ThermoFisher Scientific, Carlsbad, CA | AM7020 |  |
| Reagent | RNAeasy Mini Kit | Qiagen, Carlsbad, CA | 74104 |  |
| Equipment | Centrifuge | Eppendorf | 5425 |  |
| Equipment | Tapestation | Agilent Technologies, San Diego, CA, USA | 4200 |  |
| Equipment | Novaseq | (Illumina Inc., San Diego, CA, USA) | 6000 |  |
| Software | Trimmomatic | (1) | Ver. 0.39 | http://www.usadellab.org/cms/?page=trimmomatic |
| Software | Salmon | (2) | Ver. 1.3.0 | https://salmon.readthedocs.io |
| Software | Tximeta | (3) | Ver. 3.11 | https://www.bioconductor.org/packages/release/bioc/html/tximeta.html |
| Software | MultiQC | Stockholm University, Stockholm, Sweden (4) | Ver. 1.9 | https://multiqc.info/docs/ |
| Software | DESeq2 | (5) | Version 1.33.4 | https://www.bioconductor.org/packages/devel/bioc/vignettes/DESeq2/inst/doc/DESeq2.html |
| Software | Ingenuity Pathway Analysis | Qiagen, Carlsbad, CA | Winter 2021 | https://digitalinsights.qiagen.com |
| Software | ViSEAGO | (6) |  | https://www.bioconductor.org/packages/release/bioc/html/ViSEAGO.html |
| Database | Mouse genome |  | M29 (GRCm39) | https://www.gencodegenes.org/mouse |

**REFERENCES**

1. Bolger, A. M., Lohse, M., and Usadel, B. (2014) Trimmomatic: a flexible trimmer for Illumina sequence data. *Bioinformatics* **30**, 2114-2120

2. Patro, R., Duggal, G., Love, M. I., Irizarry, R. A., and Kingsford, C. (2017) Salmon provides fast and bias-aware quantification of transcript expression. *Nat Methods* **14**, 417-419

3. Love, M. I., Soneson, C., Hickey, P. F., Johnson, L. K., Pierce, N. T., Shepherd, L., Morgan, M., and Patro, R. (2020) Tximeta: Reference sequence checksums for provenance identification in RNA-seq. *PLoS Comput Biol* **16**, e1007664

4. Ewels, P., Magnusson, M., Lundin, S., and Käller, M. (2016) MultiQC: summarize analysis results for multiple tools and samples in a single report. *Bioinformatics* **32**, 3047-3048

5. Love, M. I., Huber, W., and Anders, S. (2014) Moderated estimation of fold change and dispersion for RNA-seq data with DESeq2. *Genome Biol* **15**, 550

6. Brionne, A., Juanchich, A., and Hennequet-Antier, C. (2019) ViSEAGO: a Bioconductor package for clustering biological functions using Gene Ontology and semantic similarity. *BioData Mining* **12**, 16
